# Generational variation and life‑course accumulation of social inequalities in all-cause mortality in Germany: a cohort-sequential study

**DOI:** 10.1186/s12939-026-02973-1

**Published:** 2026-08-04

**Authors:** Daniel Tarekegn Worede, Alfons Hollederer

**Affiliations:** 1 Research Associate at the Faculty of Human Sciences, University of Kassel, Arnold-Bode-Str 10, 34127 Kassel, Germany; 2 Professor at the Faculty of Human Sciences, University of Kassel, Arnold-Bode-Str 10, 34127 Kassel, Germany

**Keywords:** SES, Cohort, Life course, SOEP, Mortality, Germany

## Abstract

**Background:**

Social inequalities in mortality persist as a public health challenge across Europe, including Germany, despite significant health gains. Research shows a lasting social gradient in survival. However, comprehensive longitudinal evidence on cohort and life-course dynamics is limited. We examine trends in social inequalities in all-cause mortality, cohort differences, and whether cumulative low-SES exposure raises mortality risk over the life course.

**Method:**

We used longitudinal data from the German Socio-economic Panel (SOEP, 1996–2023). A cohort-sequential survival analysis included 112,037 individuals, totalling 660,480 observations and 4,974 deaths. Five birth cohorts (1925–1975) were defined. Socio-economic status (SES) was measured using education, income, and occupation, combined and modelled as both time-varying and constant exposures. Mortality was ascertained via year of death. We estimated adjusted hazard ratios (aHRs) and 95% confidence intervals (CIs) using Cox proportional hazards models with attained age as the time scale. Analyses were adjusted for sex, migration background, and marital status. Model diagnostics and sensitivity analyses were performed.

**Results:**

Mortality declined over time. However, relative social inequalities persisted. Individuals with low SES had over twice the mortality risk of those with high SES. In our life-course analysis, we found that repeated or cumulative social disadvantage across adulthood mattered more than single episodes. Each additional year spent in low SES increased mortality risk by 16% at age 50 (HR = 1.16; 95% CI: 1.11–1.22), though this effect decreased with age. We also observed generational variations: for example, low-SES individuals born in the 1956–1965 cohort (HR = 2.03, 95% CI 1.15–3.57) and 1966–1975 cohort (HR = 2.32, 95% CI 1.03–5.30) had higher mortality hazards than the 1925–1934 reference cohort. Finally, men had a higher mortality risk than women (HR 1.84, 95% CI 1.65–2.05).

**Conclusions:**

While mortality has declined, social inequality in survival persists. It accumulates over the life-course and varies by generation. These findings underscore the need for sustained, life-course policies: reducing early-life disadvantage, strengthening stable working-age, and addressing structural drivers of survival inequalities.

**Supplementary Information:**

The online version contains supplementary material available at 10.1186/s12939-026-02973-1.

## Introduction

Social inequalities in mortality remain a persistent public health challenge across Europe, including Germany, a wealthy nation with a comprehensive welfare and universal healthcare. Evidence shows that health disparities persist, even as population health improves [1–7]. The social differences in morbidity, mortality, and life expectancy are widely recognised as key indicators of structural inequality [1–3, 8]. Social or socio-economic status (hereafter, SES) is defined as an individual’s position in a social structure, typically measured by education, income, and occupational status [8–10]. These factors consistently predict survival by influencing exposure to material resources, psychosocial stressors, and health-promoting opportunities across the life course [11]. All-cause mortality is a robust indicator of population health status and serves as a key health outcome for monitoring socio-economic inequalities across social strata (GBD, [3, 12–15]).

Evidence from Germany documented an overall decline in mortality, along with the persistence of, and in some indicators, widening social inequalities in mortality [1, 3, 16]. The area-level studies found higher mortality and shorter life expectancy in socioeconomically deprived districts [17, 18]. However, the body of research is fragmented because studies use different data sources and methods. For example, some studies focus on area-level deprivation, use administrative data, while earlier SOEP analyses had short follow-ups, relied on a single SES indicator, involved few events, and covered only until the mid-2010s [3, 14, 19, 20]. Consequently, comprehensive evidence on recent mortality trends and generational inequalities remains limited [20], leaving a nearly decade-long empirical gap.

A further major remaining gap in the existing research is the limited number of studies on the cohort and life-course approach, a robust method in life-course epidemiology [19, 21]. Individuals born in different historical periods have been exposed to distinct institutional, economic, and social contexts that drive trajectories of dis/advantage over time. In Germany, successive generations entered adulthood under markedly different historical contexts and structural conditions [22, 23]. These historical shifts likely influenced the accumulation of opportunities and risks across the generations [22, 24].

In life course epidemiology, the accumulation model argues that social disadvantage exerts its strongest influence when adverse exposures, material hardship, psychosocial stress, and unequal access to resources build up across a lifespan, producing long-term health trajectories. Furthermore, this model has significant predictive power, etiological insights, and implications for health and social policies on health inequalities. (Ben-Shlomo, [11], Clouston, [21, 25–27]. Fundamental cause theory further argues that access to flexible resources- money, knowledge, power, and social capital enables advantaged groups to maintain benefits even as specific diseases and risk mechanisms change (Lieberson, 1985; [28–30]. In contrast, the age-as-leveller hypothesis proposes that social inequalities are pronounced earlier in adulthood but diminish later in life due to biological constraints, selective survival, and institutional protections [31–34]. Based on these frameworks, life‑course epidemiology has been positioned as a central approach for examining how social inequalities drive health and mortality across generations. Although cohort studies are the best approach in life-course epidemiology [35], and scholars increasingly call for applying them using longitudinal data to strengthen empirical evidence, such research remains limited in Germany [19, 36]. This gap is evident in public health research, where life-course analyses are much less common than in sociological research. Few studies have examined cohort-specific trajectories of social inequalities in mortality.

Global health frameworks also underscore that reducing social inequalities and premature mortality is a central priority, as reflected in the Sustainable Development Goals (SDG 3) and the Commission on Social Determinants of Health (CSDH) [10, 37]. These frameworks highlight that mortality inequalities are driven by social, economic, and structural determinants and require coordinated, cross-sectoral action. Within Europe, Germany contributes to the WHO Health Equity Status Report initiative, which documents persistent social gradients in mortality and life expectancy. Despite these commitments, substantial social inequalities in mortality remain.

This study analyses data from the German Socio-economic Panel (SOEP) from 1996 to 2023. It examines social inequalities in all-cause mortality across nearly three decades. Using a multidimensional SES and life‑course epidemiology approach, the study explores (1) Time trends of SES inequalities in all-cause mortality, (2) cohort variation, and (3) whether cumulative exposure to low SES increases mortality risk over the entry adult life course. By adopting cohort and life‑course approaches, the study offers new evidence on the dynamics of social inequalities in mortality in Germany.

## Methods and materials

### Study design and data source

We conducted a cohort-sequential study using data from the German Socio-economic Panel (SOEP-CORE), an ongoing annual household study initiated in 1984 that provides detailed socio-economic, demographic, and health information [38, 39]. SOEP uses a stratified multi-stage sampling design with regular refreshment samples to maintain representativeness. For this research, SOEP-CORE wave 40 (v40.1) was employed, featuring harmonised variables, standardised income imputations, and enhanced longitudinal consistency, thereby offering high-quality data for the study period [38, 39]; SOEP Group, [40].

### Study population

The analytical sample was drawn from the SOEP tracking file and included 1,446,345 observations. We included individuals aged ≥ 16 years who were interviewed between 1996 and 2023 and had valid data on education, occupation, and household income. After exclusions, 112,037 individuals contributed 660,480 observations. Participants entered the risk set at their first eligible interview and were followed until death, loss to follow-up, or administrative censoring in 2023.

### Outcome and exposure

All‑cause mortality was the primary outcome variable. Deaths during follow‑up were coded as 1, while right‑censored observations in 2023 were coded as 0. Death was ascertained using the year of death recorded in SOEP. Socio-economic status (SES) is an exposure (independent) variable in our study. To construct the SES index, education was first measured using the Comparative Analysis of Social Mobility in Industrial Nations scheme (CASMIN) and grouped into low, medium, and high categories [8]. Next, occupational status was measured by the International Socio-economic Index of Occupational Status (ISEI) [41]. Then, household income was equivalized using the Organisation for Economic Co-operation and Development (OECD) modified scale based on multiply imputed monthly net income. Each component (education, occupational status, income) was scored on a scale of 1 to 7. The scores were summed (range: 3–21) (See Supplementary Table S1 A-E). Finally, the total SES index was categorised into quantiles as low (the lowest 20%), middle (the middle 60%), and high (the highest 20%) SES groups [8, 42].

### Covariates

All models were adjusted for sex (male, female), age (years), migration background (non-migrant, migrant), and marital status (married, separated or divorced, widowed, single). Sensitivity analyses additionally included self-rated health (poor and good) and overall life satisfaction (low, medium, high) to examine potential mediation pathways [42, 43].

In the life course analysis, we used attained age (in years) as the time scale. We also defined sequential birth-cohort groups to assess mortality variations across generations at the observed ages. These cohorts reflect broad historical contexts: social, economic, and policy conditions. Such factors shape cumulative exposure to advantages and disadvantages in the life course. We adapted Simonson et al.,‘s [23] study to group individuals into five cohort groups: 1925–1934, 1935–1945, 1946–1955, 1956–1965, and 1966–1975 [23]. This grouping allows us to analyse age-patterned variations across cohorts. Furthermore, we defined three observation periods—1996–2005, 2006–2014, and 2015–2023 to assess temporal trends.

### Analytical approach

All analyses were conducted using Stata 18 (StataCorp). Data preparation began with the SOEP longitudinal tracking file (ppathl), which contains all individuals ever observed in the panel. Three criteria are used for inclusion of individuals in the sample: (1) be aged 16 years or older, (2) reside in a private household at the time of the survey, and (3) have completed a successful interview between 1996 and 2023. Individuals were linked across survey waves using personal identifiers (pid) and survey year (syear). SES and covariates were merged from the pgen and pequiv files (one‑to‑one merges by pid and syear), and equivalised net household income was added from the household dataset (hgen) using one‑to‑many merges by hid and syear. Only relevant information for SES construction and covariates was kept. We applied the longitudinal weights provided by SOEP to compensate for unequal sampling, nonresponse, and panel attrition, thereby improving the representativeness of the analytical sample. Cluster‑robust standard errors were used to account for repeated observations within individuals.

Descriptive statistics were used to summarise the study variables in counts and proportions (%), and person‑years mortality rates per 1,000 person‑years. We then generated Kaplan–Meier survival curves and applied log‑rank tests to evaluate overall and group‑specific differences in mortality risk. Then, hazard ratios were estimated using Cox proportional hazards models [44] via the Stata stcox command, computing relative risks of all‑cause mortality as hazard ratios (HRs) with 95% confidence intervals(CI); results were considered significant at *P* < 0.05. Survival time data were declared, and attained age was used as the underlying time scale to account for age-related mortality risk [45, 46].

We fitted three complementary Cox proportional hazards models to examine temporal trends, cohort variation, and life‑course social inequalities in mortality, estimating hazard ratios. Model 1: to estimate the extent and temporal trends of SES inequalities, we fitted a main model including SES and study period, followed by SES × period interaction model. Model 2: to investigate cohort variations, we applied the same two-step approach: an SES–cohort main model and SES × cohort interaction model to evaluate generational variation [47]. Period‑ and cohort‑specific SES variations were also computed using linear combinations of main and interaction terms. Model 3: to analyse life‑course cumulative social disadvantage, we modelled cumulative low SES as a continuous variable with a time‑varying effect, as it violated the proportional hazards (PH) assumptions. Three models were adjusted for sex, migration background, and marital status, and Sensitivity analyses additionally adjusted for self‑rated health and overall life satisfaction to examine potential mediation pathways for each interaction model.

Hazard ratios (HRs) were obtained by exponentiating the estimated coefficients. PH assumptions were tested using Schoenfeld residuals (global goodness-of-fit) and graphical diagnostics. Furthermore, the significance of the interaction was tested using a likelihood-ratio test with the Stata (lrtest) command.

## Result

### Descriptive characteristics

The analytic sample included 112,037 individuals contributing 660,480 observations and 684,295 person‑years of follow‑up. Participants were followed for an average of 6.1 years, entering at a mean age of 40 and exiting at 46 years. Women contributed slightly more person‑time than men (52.5% vs. 47.5%), and 4,974 deaths (4.44%) occurred during follow‑up. Men showed a higher crude mortality rate than women (8.4 vs. 6.2 deaths per 1,000 person‑years). Clear social gradients were evident: mortality was highest in the low‑SES group (7.1 per 1,000 person‑years) and lowest in the high‑SES group (2.3 per 1,000). Similar patterns appeared across occupational class, education, and equivalised household income. Individuals without a migration background had higher mortality (8.4 per 1,000) than migrants (3.8 per 1,000). Full descriptive results for all variables are presented in (Supplementary Table S2A-B).

### Kaplan–meier survival gradients and log-rank tests

**Fig. 1:**
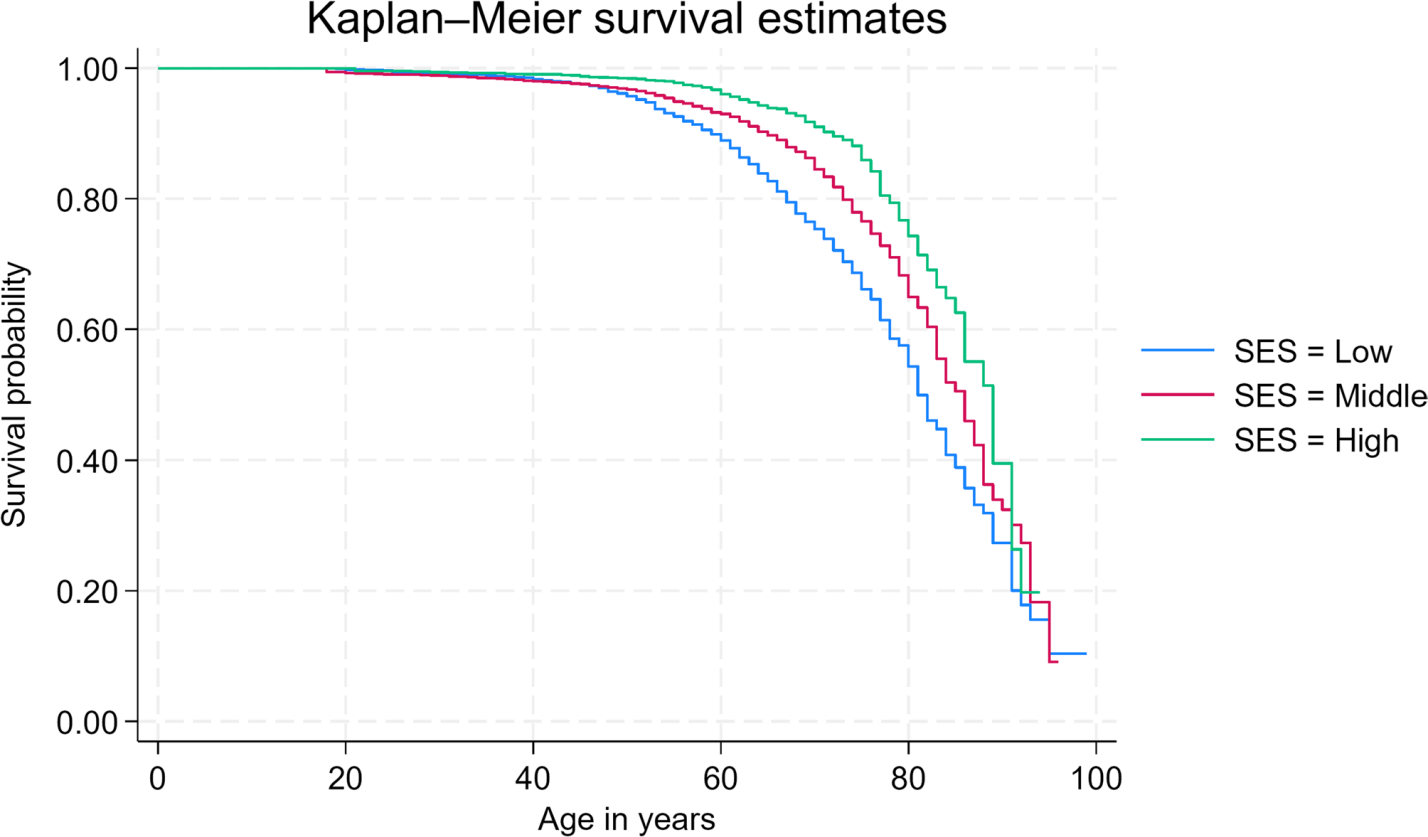
The Kaplan–Meier Survival Curve (SES strata), SOEP 1996–2023

Kaplan–Meier curves showed the expected age‑related decline in survival, with probabilities remaining high through early adulthood and decreasing more steeply after age 70 (Supplementary Figure S1). Clear social gradients were evident: the low‑SES group experienced an earlier and sharper drop in survival than the middle‑ and high‑SES groups, which maintained higher survival probabilities into older age (Fig. 1). The log‑rank test confirmed significant differences across SES groups (χ²(2) = 143.08, *p* < 0.001).

**Fig. 2:**
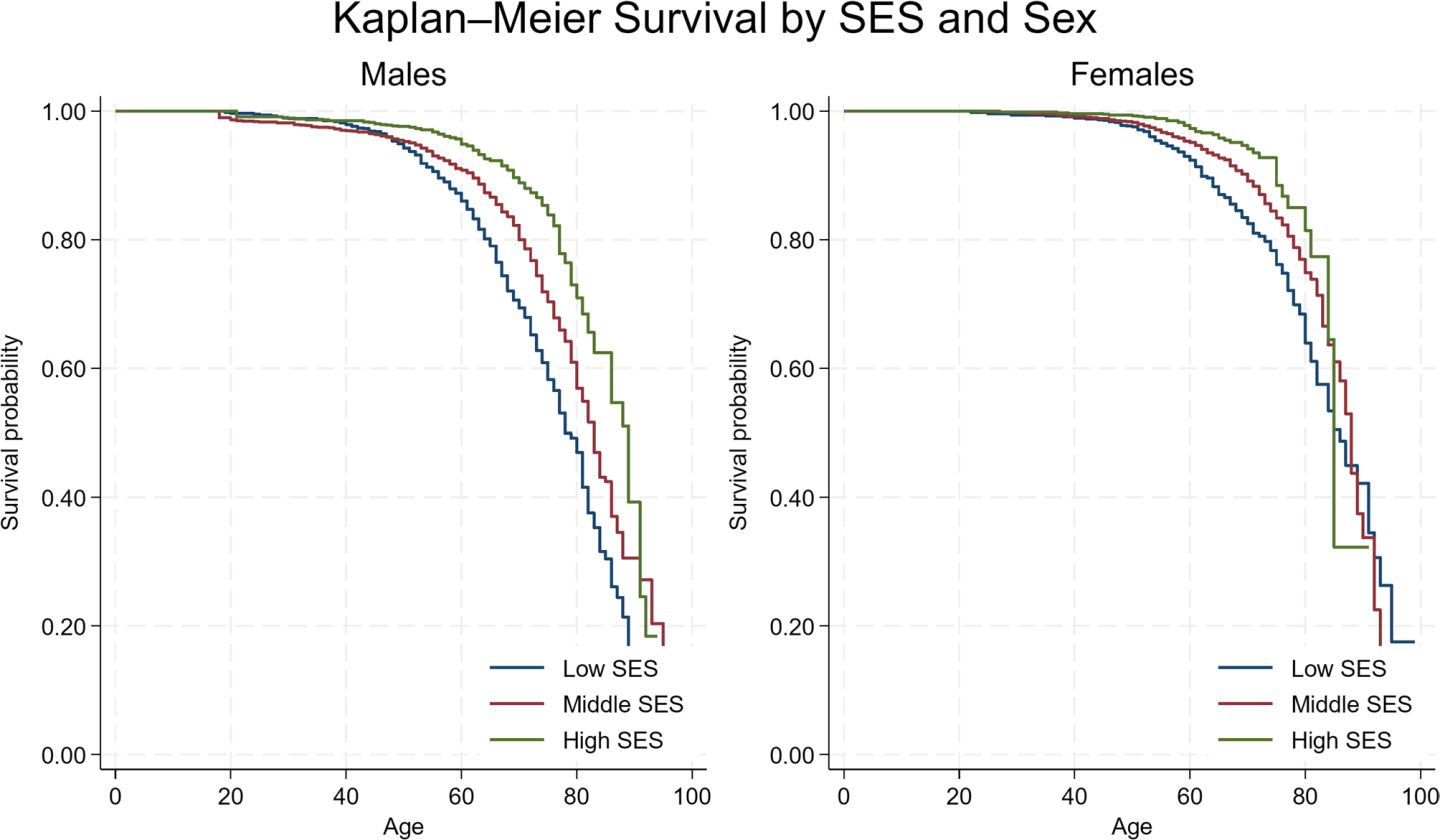
The Kaplan–Meier Survival Curve (SES with sex), SOEP 1996–2023

Sex‑specific survival curves revealed further inequalities. Males experienced a sharper decline in survival between ages 60 and 70, whereas females maintained higher survival rates into their seventies and eighties (Fig. 2). Even females in the middle‑SES group outlived men in the high‑SES group, underscoring the combined influence of gender and social status on mortality risk (Supplementary Figures S2 and S3). These unadjusted nonparametric survival analyses highlight the magnitude and existence of social and gender inequalities in survival. Furthermore, individuals who are separated or widowed and single individuals showed a higher mortality risk compared to married individuals. Between migrants and non-migrants, no significant differences in mortality risks are observed in the unadjusted analysis.

### Time trends of SES inequalities in mortality across study periods

In the primary analysis, the main model indicated that individuals with low SES had more than double the mortality risk compared with those with high SES (HR = 2.34, 95% CI 1.96–2.79). Middle-SES individuals also had a high mortality risk (HR = 1.64, 95% CI 1.39–1.93). Mortality declined over time: hazards were 32% lower in 2006–2014 (HR = 0.68, 95% CI 0.60–0.76) and 50% lower in 2015–2023 (HR = 0.50, 95% CI 0.43–0.57) compared to 1996–2005 (Table 1).

**Table 1:** Main effects of SES and Period, and SES x Period interaction model (Model 1), SOEP 1996–2023

Variable	Category	Main‑Effects Model HR (95% CI), *p*‑value	Interaction Model HR (95% CI), *p*‑value
*SES* *High (ref)*	*Low*	*2.34 (1.96–2.79)*,p < 0.001*	*2.31 (1.67–3.18)*,p < 0.001*
	*Middle*	*1.64 (1.39–1.93)*,p < 0.001*	*1.71 (1.25–2.36)*,p = 0.001*
*Period*	*1996–2005 (ref)*	*–*	*–*
	*2006–2014*	*0.68 (0.60–0.76)*,p < 0.001*	*0.72 (0.49–1.06)*,*p = 0.093*
	*2015–2023*	*0.50 (0.43–0.57)*,p < 0.001*	*0.48 (0.33–0.72)*,p < 0.001*
*SES × Period*	*Low × 2006–2014*	*–*	*0.93 (0.61–1.42)*,*p = 0.727*
	*Low × 2015–2023*	*–*	*1.26 (0.80–1.99)*,*p = 0.313*
	*Middle × 2006–2014*	*–*	*0.93 (0.61–1.41)*,*p = 0.732*
	*Middle × 2015–2023*	*–*	*0.92 (0.60–1.42)*,*p = 0.707*
*Sex* *Female (ref)*	*Male*	*1.84 (1.65–2.04)*,*p < 0.001*	*1.83 (1.65–2.04)*,p < 0.001*
*Marital Status* *Married (ref)*	*Separated/Divorced*	*1.36 (1.18–1.57)*,*p < 0.001*	*1.36 (1.17–1.57)*,p < 0.001*
	*Widowed*	*1.17 (0.99–1.38)*,*p = 0.072*	*1.17 (0.99–1.38)*,*p = 0.068*
	*Single*	*1.78 (1.48–2.16)*,*p < 0.001*	*1.79 (1.48–2.16)*,p < 0.001*
*Migback* *non-mig (ref)*	*Migrant*	*0.97 (0.84–1.11)*,*p = 0.636*	*0.97 (0.84–1.11)*,*p = 0.615*

* P-value Significan at *P* < 0.05, ref= reference group, migback= migration background, non-mig = non-migrant

Building on these findings, the interaction model showed that low SES was strongly linked to higher mortality (HR = 2.31, 95% CI 1.67–3.18). Middle SES was also elevated (HR = 1.71, 95% CI 1.25–2.36) compared to the reference period (1996–2005). Although absolute mortality fell over subsequent periods, none of the SES × period interaction terms reached statistical significance (all *p* > 0.30). This suggests that relative SES inequalities remained largely persistent across periods.

Male individuals experienced higher mortality than female individuals. Those who were single or divorced faced higher mortality risks compared to married individuals. Migration background did not show an association with mortality (Table 1).

The proportional hazards assumption was met (global Schoenfeld test, χ²(13) = 15.45, *p* = 0.28). Multicollinearity was minimal (mean VIF = 1.30). Overall, these findings indicate strong and persistent SES inequalities in all-cause mortality across nearly three decades, despite notable improvements in population survival.

### SES inequalities in all-cause mortality across birth cohorts

We have observed different mortality patterns among birth cohorts. In the main effects model, individuals with low SES had a higher mortality risk than the high SES reference group (HR = 2.42, 95% CI 2.04–2.95, *p* < 0.001). Individuals with middle SES also showed higher mortality risk (HR = 1.65, 95% CI 1.39–1.96, *p* < 0.001).

In addition to the observed SES variation, absolute mortality hazards declined in successive cohort groups. Relative to the 1925–1934 cohort, later cohorts saw progressively lower mortality hazards, exemplified by an HR of 0.25 (95% CI 0.18–0.35, *p* < 0.001) for individuals born in 1966–1975. The proportional hazards assumption was met (global Schoenfeld *p* = 0.56).

In the interaction model, we also found significant variations among cohort groups. The association between low SES and mortality risk increased across cohort groups. For example, low-SES individuals in the 1956–1965 cohort had a higher mortality hazard than those in the 1925–1934 reference cohort (HR = 2.03, 95% CI 1.15–3.57, *p* = 0.014). This magnitude increased further in the 1966–1975 cohort (HR = 2.32, 95% CI 1.03–5.30, *p* = 0.047), indicating a widening inequality across successive cohorts.

Similarly, for middle SES, a significant interaction effect between SES and birth cohort was observed in the 1966–1975 cohort group (HR = 2.23, 95% CI 1.04–4.79, *p* = 0.039) (Table 2). However, the wide confidence intervals for both low and middle SES 1966–1975 cohorts reflect the relatively small number of deaths in the youngest cohort, and the estimates should therefore be interpreted with caution. Furthermore, in our analysis, we did not disentangle the cohort effect from the age effect; the observed variation is based on age-structured cohort patterns.

**Table 2:** Main effects of SES and Cohort, and SES x Cohort interaction model (Model 2), SOEP 1996–2023

Variable	Category	Main‑Effects Model HR (95% CI), *p*‑value	Interaction Model HR (95% CI), *p*‑value
*SES (high (ref)*	*Low*	*2.42 (2.04–2.95)*,p < 0.001*	*1.74 (1.20–2.50)*,p < 0.004*
	*Middle*	*1.65 (1.39–1.96)*,p < 0.001*	*1.25 (0.86–1.85)*,*p = 0.241*
*Cohort* *1925–1934 (ref)*	*1935–1945*	*0.69(0.59–0.82)*,*p < 0.001*	*0.57 (0.36–0.89)*,p < 0.014*
	*1946–1955*	*0.46 (0.37–0.56)*,p < 0.001*	*0.30 (0.18–0.50)*,p < 0.014*
	*1956–1965*	*0.34 (0.26–0.43)*,p < 0.001*	*0.23 (0.14–0.40)*,p < 0.001*
	*1966–1975*	*0.25 (0.18–0.35)*,p < 0.001*	*0.12 (0.06–0.26)*,p < 0.001*
*SES × Cohort*	*Low ×1935–1945*		*1.21 (0.76–1.95)*,*p = 0.445*
	*Low ×1946–1955*	*–*	*1.62 (0.94–2.79)*,*p = 0.082*
	*Low ×1956–1965*	*–*	*2.03 (1.15–3.57)*,p = 0.014*
	*Low× 1966–1975*	*–*	*2.32 (1.03–5.3)*,p = 0.047*
	*Middle × 1935–1945*	*–*	*1.24 (0.73–2.21)*,*p = 0.386*
	*Middle × 1946–1955*		*1.48 (0.87–2.51)*,*p = 0.143*
	*Middle × 1956–1965*	*–*	*1.27 (0.73–1.81)*,*p = 0.392*
	*Middle × 1966–1975*	*–*	*2.23 (1.04–4.79)*,p = 0.039*
*Sex* *Female (ref)*	*Male*	*1.84 (1.65–2.05)*,p < 0.001*	*1.83 (1.64–2.04)*,p < 0.001*
*Marital Status* *Married (ref)*	*Separated/Divorced*	*1.35 (1.17–1.57)*,p < 0.001*	*1.34 (1.16–1.56)*,p < 0.001*
	*Widowed*	*1.23 (1.03–1.45)*,*p = 0.019*	*1.24 (1.04–1.46)*,p = 0.014*
	*Single*	*1.76 (1.43–2.15)*,p < 0.001*	*1.76 (1.44–2.15)*,p < 0.001*
*Migback non-mig (ref)*	*Migrant*	*0.96 (0.83–1.11)*,*p = 0.602*	*0.96 (0.83–1.10)*,*p = 0.552*

* P-value Significant *P* < 0.05, ref= reference group, migback= migration background, non-mig = non-migrant

Since we found significant interaction effects in the interaction model, we conducted cohort-specific analyses. We estimated hazard ratios from the combined main-effects and interaction model. The hazard ratio for low versus high SES increased across cohorts: 1.74 in 1925–1934, 2.10 in 1935–1945, 2.82 in 1946–1955, 3.53 in 1956–1965, and 4.04 in 1966–1975. This suggests a progressively widening social gradient in survival. Supplementary Table S4 and Supplementary Figure S4 also show this pattern.

### Life course accumulation of social inequalities in all-cause mortality

We fitted a model centred on age at 50 as a reference point (age = 0), with younger ages taking negative values and older ages positive values. Using this specification, we found a strong association between cumulative social disadvantage and mortality risk. At age 50, each additional year spent in low SES was associated with a 16% higher hazard of death (HR = 1.16, 95% CI: 1.11–1.22).

The time-varying component indicated that the association between cumulative social disadvantage and mortality risk became weaker with increasing age. Specifically, the interaction HR of 0.998 (*p* < 0.001) suggests that, for each additional year beyond age 50, the excess mortality risk associated with additional years in low SES declined slightly. While the effect size is modest, this trend suggests that cumulative social disadvantage has a stronger effect on mortality in early and mid-adulthood, with this influence gradually lessening in later adulthood.

In addition to the primary relationships observed, covariates also showed expected associations: men had a substantially higher mortality risk than women; individuals who were separated/divorced, widowed, or single had higher mortality than those who were married. In contrast, a migration background was not significantly associated with mortality in this model (Table 3).

**Table 3:** Association of cumulative Low SES and mortality risk in the Life Course (Model 3), SOEP 1996–2023

Variable	Category	HR	95% CI	*p*-value
*Cumulative low-SES*	*Continuous*	*1.16*	*1.11–1.22*	*0.000*
*Sex Female (ref)*	*Male*	*1.57*	*1.48–1.67*	*0.000*
*Marital status Married (ref)*	*Separated/Divorced*	*1.33*	*1.21–1.46*	*0.000*
	*Widowed*	*1.44*	*1.34–1.56*	*0.000*
	*Single*	*1.55*	*1.37–1.75*	*0.000*
*Migback-Non-mig(ref)*	*Migrant*	*1.06*	*0.97–1.15*	*0.183*
*Time-varying effect*	*cum_lowSES × age*	*0.99*	*0.99–0.99*	*0.000*

Low-SES = low socio-economic status; ref = reference category; migback = migration background; non-mig = non-migrant; CI = confidence interval; HR = Hazard Ratio

**Fig. 3:**
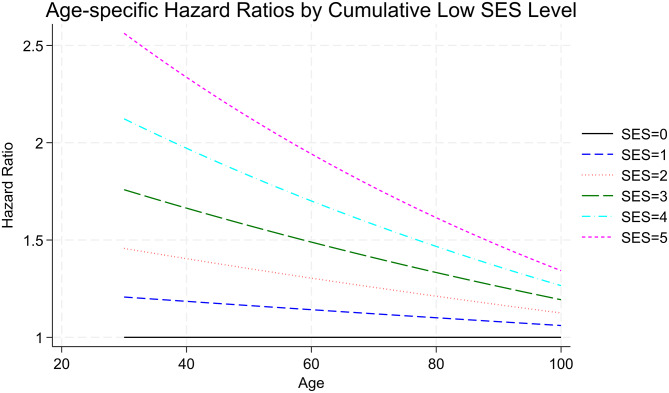
shows the age-specific hazard ratios by cumulative low SES exposure level (0–5 scale), SOEP 1996–2023

Figure 3 displays the predicted age-specific hazard ratios for mortality, categorised by different levels of cumulative exposure to social disadvantage. The reference group includes individuals who were never exposed to low SES (cumulative SES = 0, indicating either middle or higher SES value). Cumulative low SES is defined as the number of observation periods in which individuals experienced low SES. This number ranges from 0 to 5, with higher values indicating greater accumulation of social disadvantage over the life course.

The figure reveals a clear, graded association between cumulative low SES and mortality risk. At every age, individuals with more years of low SES have higher hazard ratios than those with fewer or no years of social disadvantage. Individuals with higher (longer) exposure levels show a higher mortality risk throughout all ages. This pattern illustrates strong cumulative social disadvantage. Across all exposure levels, hazard ratios decline gradually with age. Importantly, even though the relative differences narrow, the social gradient remains evident as people age.

### Sensitivity analysis

Sensitivity analyses were conducted to assess the robustness of the SES–mortality associations to additional adjustment for self-rated health and overall life satisfaction, two indicators that may partly capture baseline health status and subjective well-being. As expected, inclusion of these variables attenuated the estimated SES effects across all three analytical models. The overall pattern of results remained consistent with the main analyses, indicating that the findings are robust to alternative model specifications (see supplementary Tables S4, S5, S6 and S7).

## Discussion

We conducted a comprehensive longitudinal life-course epidemiological analysis of social inequalities in all-cause mortality across the adult life course in Germany over nearly three decades, integrating life-course and cohort approaches. This study advances existing evidence by investigating the life-course accumulation of social inequalities, cohort variations, and time trends in all-cause mortality. We found a strong association between social inequality and all-cause mortality, consistent with prior evidence. Importantly, this study adds three new findings. First, the social disadvantage that is repeated or accumulates across the adult life course matters more than single episodes. Each episode of disadvantage increased the hazard of all-cause mortality. Second, as people age, the association becomes weakened but persists throughout the lifespan, indicating a strong association in early to middle adulthood. Third, we observed that birth cohorts differ in the strength and pattern of the association between social inequality and all-cause mortality, suggesting that the effect of social inequalities varies across generations.

### Social Inequalities in all-cause mortality across birth cohorts

We observed differences in the strength of the association between SES and all-cause mortality across birth cohort groups. These variations reflect age-structured cohort patterns rather than isolated cohort effects, given the inherent interdependence of age, period, and cohort.

In the interaction model, the excess mortality risk associated with low-SES was significantly higher in the 1956–1965 and 1966–1975 cohorts compared with the 1925–1934 reference cohort. Low-SES individuals born in 1956–1965 had approximately twice the mortality hazard of their high-SES counterparts in the reference cohort, while those born in 1966–1975 had a 2.3-fold higher hazard. A similar pattern was observed in the youngest cohort for middle SES, with the 1966–1975 group showing a 2.23-fold higher hazard relative to the reference cohort.

Across cohorts, we observed a decline in absolute mortality. This might reflect differences in age structure: older cohorts have more exposure time and more deaths, while younger cohorts have lower mortality due to younger age. The declines might also signal long-term improvements in healthcare and living standards, as well as reductions in infectious disease mortality. This explanation is supported by global burden of disease studies, which show that age-standardised mortality has been decreasing since the 1950s (GBD, [12]). However, while absolute mortality declined, the magnitude of relative social inequalities in mortality hazard grew compared to older cohort groups. Both the interaction model and cohort-specific estimates showed this pattern. These rising gradients align with broader social and economic changes affecting younger generations. Changes in the labour market, education, and macroeconomic shocks have shifted social resources and opportunities, possibly driving these differences ([15, 22]; Felix & [48]). Behavioural risk factors also follow cohort-specific trajectories. For example, older East German women smoke less, whereas younger women smoke more after reunification [24]. Disease-stage theory further posits that inequalities widen when new medical technologies or preventive measures diffuse unevenly across social groups [25, 49]. As chronic diseases have replaced infectious diseases, health behaviours and access to preventive strategies have become increasingly stratified by social class [29]. Adolescence and early adulthood—key periods for building long-term risks—are especially sensitive to social disadvantage. Social status shapes unhealthy diet, obesity, and smoking patterns at this life stage, which are major risk factors for cardiovascular disease and mortality [50].

International evidence shows similar trends. British cohort studies report widening social inequalities in survival among younger generations [47]. A multi-country European analysis found rising alcohol-related mortality in successive cohorts, with increasing gradients among recent generations. Other patterns, like road traffic and suicide mortality, are similar [12, 49].

Taken together, these findings suggest that the observed cohort variations in the association likely reflect age differences and long-term changes in disease patterns, working and living conditions, and the distribution of social and behavioural resources.

### Life-course accumulation of social inequalities in all-cause mortality

The life-course analysis indicates a clear cumulative effect of social disadvantage on mortality. Each additional year spent in low socio-economic status (SES) increases mortality risk, with a stronger association observed in mid-adulthood. At age 50, an extra year in low SES is associated with a 16% higher mortality hazard. This aligns with earlier findings from Lampert et al., [3], who also observed higher mortality risks among individuals younger than 50 compared with those aged 50 or older using SOEP data. However, their study relied solely on income comparisons between low-income and high-income groups, whereas our analysis incorporates repeated exposure to disadvantage using time-varying measures and interaction models.

Furthermore, evidence reinforces the link between social disadvantage and mortality in Germany, even though the studies are not comparable. The Gutenberg Health Study reported that social disadvantage doubles the risk of mortality and cardiovascular disease [51]. Area-level deprivation studies similarly show elevated premature mortality in highly disadvantaged areas [2]. Yet these studies focus on cross-sectional or area-based measures rather than on the longitudinal accumulation of disadvantage, which is central to our approach.

International research shows both similarities and contrasts with the age pattern we observe. Consistent with our findings, several longitudinal studies show that disadvantage in both childhood and adulthood increases the risk of chronic disease and mortality [52, 53]. Life-course epidemiology conceptualises social conditions as cumulative exposures that shape health trajectories; material hardship, psychosocial stress, and constrained resources during working age may become entrenched, elevating later-life mortality risk [11, 54, 55]. However, Pudrovska (2014) found widening inequalities with age in the United States, with annual increases in the mortality gap of roughly 10% for women and 8.8% for men. Such discrepancies likely reflect methodological differences, population characteristics, and cross-national variation in welfare and healthcare systems. In our study, baseline health and well-being partially attenuated the association between disadvantage and mortality, whereas Pudrovska (2014) found that these factors had limited explanatory value. Germany’s comprehensive health coverage and strong welfare institutions may help reduce late-life inequalities, contributing to the narrower age-related gradient observed in our results.

Furthermore, our time-varying analysis reveals a modest reduction in SES differences at older ages. After age 50, the association between social disadvantage and mortality weakens slightly, but this does not necessarily reflect a true “age-as-leveller” process. Instead, socio-economic inequalities persist across the life course, though they are most pronounced in early and mid-adulthood. Selective survival—where individuals with more favourable genetic, social, and environmental resources are more likely to reach older ages—likely contributes to the narrowing of inequalities in later life [56]. Lifestyle-related behavioural factors, including smoking and drinking high alcohol, might also contribute to variations among individuals. Evidence from Germany supports this interpretation: mortality inequalities are larger below age 50 [3], and educational health inequalities decline with age [57]. Because age captures both life-stage and cohort effects, differences may reflect generational experiences as well as biological ageing [58].

### Temporal trends of social inequality in all-cause mortality

Trend analyses showed a decline in overall mortality over the study period. However, substantial inequalities persisted. Individuals with low-SES had more than twice the mortality risk compared to those with high SES (HR = 2.34, 95% CI 1.96–2.79). Notably, the trend analysis included the COVID-19 pandemic period, but no significant interaction between SES and period was observed. Previous SOEP-based research also reported persistent social gradients in mortality with no reduction from 1992 to 2016, with higher risks among men [3]. Extending previous SOEP findings into the 2020s, our study shows that inequalities persist. Other studies reinforce these disparities. For example, Grigoriev et al., [59] used large-scale pension data and reported significant income-related mortality inequalities [59]. Similarly, an area-level study found declines in mortality but persistent social inequalities [1, 15, 60, 61].

This pattern is also evident internationally. Research across Europe has revealed that while mortality has decreased since the 1990s, relative social inequalities have persisted or even increased [5, 6, 62, 63]. Together, these patterns suggest that major gains in population health can occur even as social gradients in mortality persist or widen.

Across all models, gender inequality was substantial: men had higher mortality risks than women (aHR 1.84, 95% CI 1.65–2.05, *p* < 0.001). Notably, this risk is higher than previously reported, where men with low income (< 60% of the mean) had a hazard ratio of 1.70 compared to women [3]. The observed disparity is likely driven by gender differences in health behaviours and occupational exposures—men show higher rates of tobacco and alcohol use, lower preventive healthcare uptake, and greater engagement in hazardous or physically demanding work. Employment-related research bolsters this explanation: studies indicate unemployment and precarious jobs are strongly associated with adverse health outcomes, including mortality [64, 65], and men disproportionately take on high-risk occupations in Germany. Thus, the combination of behavioural and structural factors may explain the gendered mortality gradient seen in this study.

Overall, these findings suggest that cohort differences may lead to more varied mortality trends as social and economic conditions change. Notably, life-course data show that long-term social disadvantage increases mortality risk, particularly during working age. While mortality has decreased in recent decades, social inequalities in survival have remained largely unchanged, highlighting that these inequalities result from disadvantages that build up over time.

### Strengths and limitations

This study has several important strengths. It draws on the German SOEP, a large, nationally representative, longitudinal dataset, with nearly 30 years of follow-up and ongoing data quality improvements. This enables a robust study of trends, cohort differences, and accumulation over the life course. By adopting a life-course epidemiological perspective, we examine health at different ages and stages. The study goes beyond short-term analyses to examine how social disadvantage is linked to mortality risk over time.

Methodologically, this study extends previous SOEP-based research by adding interaction terms to standard Cox proportional hazards models. This allows us to study age-dependent and cohort-specific variations in the association. We provide a nuanced understanding of how social inequalities in mortality shift over time, across generations, and life courses. Modelling cumulative SES exposure and time-varying effects offers quantitative insight into the dynamic, age-dependent nature of disadvantage.

Several limitations should be considered. First, the youngest cohort (1966–1975) had relatively few mortality events. This reduced statistical power and precision for this group. Findings for this cohort should be interpreted with caution. Second, residual confounding cannot be fully excluded. Information on early-life conditions, health behaviours, and environmental exposures is incomplete. Sensitivity analyses adjusting for psychosocial and health-related factors yielded consistent results. This supports the robustness of the main findings.

Furthermore, individuals enter the panel at different ages, making it difficult to obtain comparable age ranges across birth cohorts. This especially affects mortality cohort studies, where the number of deaths is limited. Age and cohort effects cannot be fully disentangled, so observed differences should be interpreted as age-structured patterns across birth groups, not as pure cohort effects. We used age-structured cohort groups to reflect historical contexts, but these groups do not provide full comparability across ages.

We used a composite SES indicator to capture multidimensional social status and cumulative social disadvantage in a simple, policy-relevant way. This approach helps monitor social inequalities in health and mortality. It also avoids debates about which SES indicator best captures differences. However, this composite approach may mask important distinctions between domains such as education, income, and occupation.

Finally, survey-based mortality data face challenges, as the SOEP typically reports lower mortality rates than official statistics, partly because household members or relatives may voluntarily report, leading to under-ascertainment and conservative absolute estimates. There is no strong evidence that this under-ascertainment systematically affects SES groups, so the study’s relative measures remain robust. All-cause mortality did not indicate cause-specific mortality inequality, though it remains a robust indicator of population health status.

## Conclusion, policy and research implication

While absolute mortality has decreased, social inequalities in mortality in Germany persist, varying across generations and life courses. Repeated social disadvantage increases the mortality hazard for each episode. As individuals aged, the association weakened. Notably, the strong association was observed in mid-adulthood, reflecting cumulative social disadvantage.

Building on these insights, the findings show a need for sustained, targeted life-course interventions. Policies must: (1) reduce early-life disadvantages, (2) ensure stable employment, and (3) break down educational stratification. The cumulative SES disadvantage underscores the need for long-term, life-course strategies rather than short-term ones.

To advance mortality research and promote equity in health, we propose three key actions: (1) improving the quality and completeness of SOEP mortality data, (2) integrating SOEP with additional healthcare and administrative datasets, and (3) creating a national mortality registry that includes detailed socio-economic information.

Looking ahead, future research must prioritise examining causal drivers of social inequalities in health and mortality. Specifically, research should identify the causal contributions of the major behavioural risks, work-related factors, healthcare access barriers, health technology uptake, and sources of psychosocial stress to mortality. As economic volatility, population ageing, and climate change risks exacerbate social inequalities in mortality, establishing clear research priorities using a robust causal life-course approach supported by machine learning models and continuous monitoring is essential.

## Supplementary Information

Below is the link to the electronic supplementary material.

Supplementary Material 1

Supplementary Material 2

## Data Availability

The SOEP is available for non-commercial research under strict data protection. This study uses fully anonymised secondary data collected with informed consent and in line with the German data protection law. German Institute for Economic Research (DIW Berlin) granted data access under a Scientific Use File contract (registration number 7310). Data can be accessed at: https://www.diw.de/en/diw_01.c.678568.en/research_data_center_soep.html. As all analyses use anonymised secondary data and no new data is collected, additional ethical approval is not required.
