# Supplementary material for: Generational variation and life‑course accumulation of social inequalities in all-cause mortality in Germany: a cohort-sequential study
Source: Int J Equity Health. 2026 Aug 4;25:185. doi: 10.1186/s12939-026-02973-1 (PMC13445823; doi:10.1186/s12939-026-02973-1)
Supplement: Supplementary file 1 [file 12939_2026_2973_MOESM1_ESM.docx]

**Generational variations and Life‑Course Accumulation of Social Inequalities in Mortality in Germany**

**Supplementary file**

**Table of contents**

| Description | Page |
| --- | --- |
| 1. Method | 1 |
| **Supplementary Table S1 A to E**: Construction of SES using Education, Occupational status and net monthly household income | 1 |
| 2. Result | 3 |
| **Supplementary Table S2**: *Crude mortality rates, person‑time at risk, and survival percentiles by demographic and socioeconomic characteristics, SOEP 1996–2023* | 3 |
| **Supplementary Figure S1**: The Kaplan–Meier Survival Overall Curve | 5 |
| **Supplementary Figure S2**: The Kaplan–Meier Survival Curve SES with Gender | 5 |
| **Supplementary Figure S3**: The Kaplan–Meier Survival curve for Cohorts | 6 |
| **Supplementary Table S3**. Cohort‑specific SES–mortality association (HR, 95% CI, p‑value) | 6 |
| **Supplementary Figure S4**: Cohort‑specific HR ratio low versus High SES | 6 |
| **Supplementary Table S4**: Sensitivity Analysis: SES x Period Cox Regression adjusted with Self‑Rated Health, Life Satisfaction | 8 |
| **Supplementary Table S5**. Sensitivity analysis: SES × cohort model adjusted for self‑rated health and life satisfaction | 8 |
| **Supplementary Table S6.** Sensitivity analysis: cumulative low SES adjusted for self-rated health and overall life satisfaction |  |
| **Supplementary Figure S5**: Cohort‑specific HR ratio low versus High SES | 9 |
| Detailed Sensitivity Analysis Report | 9 |

1. **Method section**

**Exposure Variable: SES**

**Supplementary Table S1 A to E: Construction of SES using Education, Occupational status and net monthly household income**

The SES construction follows the original Lamperts et al. (2013 classification, and it is as follows

A. EDUCATION

| Education level CASMIN | Description | SES point |
| --- | --- | --- |
| 1a,1b | No formal education/primary | 1 |
| 1c | Lower secondary | 2 |
| 2a | intermediate secondary | 3 |
| 2b | General maturity certificate (Abitur) | 4 |
| 2c | Vocational quaölification | 5 |
| 3a | Higher education /technical college | 6 |
| 3b | University degree | 7 |

B. **Occupation: First, we harmonised isei88 (1996-2017) and isei08 (2018-2023) (Ganzenboom, 1992)**

| Occupational status ISEI88 &ISEI08 | SES point |
| --- | --- |
| Noccupation | 1 |
| 11.56-25 | 2 |
| 26-35 | 3 |
| 36-45 | 4 |
| 46-55 | 5 |
| 56-65 | 6 |
| 66-90 | 7 |

C. **Net household (HH) Income**: The 3^rd^ important factor, income, is also classified into 7 categories based on the national income distribution, following the Lampert et al. (2013 classification for comparability, reproduction, future studies, monitoring, and reporting. We exclude extreme values.

| Income Percentile | SES point |
| --- | --- |
| 1-9 | 1 |
| 10-20 | 2 |
| 21-40 | 3 |
| 41-60 | 4 |
| 60-80 | 5 |
| 81-90 | 6 |
| >90 | 7 |

D. **Summed SES**

Using the summing of education, occupational status and net monthly HH income.

**SES=Education+ Occupation+ Income**: Then the indicators are scored from **3 to 21**, and this score is classified into three major SES indices by putting together

E. **Quantiles**

Robert Kock Institute Income defines five equally sized income groupings (20% each), standardised into five quantiles (Q1-Q5) (Lampert et al.,2013)

| Quantiles | SES point |
| --- | --- |
| 1^st^ (lowest) | 3.0-7.7 |
| 2^nd^ | 7.8-9.6 |
| 3^rd^ | 9.7-11.7 |
| 4^th^ | 11.8-14.1 |
| 5^th^ (Highest) | 14.2-21.0 |

**SES-Index as Categorical:** Using the total SES score, the distribution is therefore divided into:

1. **Low SES=1^st^ quantile =20%**

Low SES = 3.0-7.7

2. **Middle SES= (2^nd^ 3^rd^ 4^th^ quantiles^)^ =60%**

Middle SES=7.8-14.1

3. **High SES = (5^th^ Quantile) =20%**

High SES=14.2-21.0

In our analysis, we estimated models using both continuous and categorical SES measures, but we ultimately reported results based on the categorical version. While the continuous SES indicator is more suitable for modelling, the three‑level SES grouping is easier to interpret and communicate. It also provides a clear picture of inequality, especially when contrasting the lowest 20% with the highest 20% of the population. This makes the categorical measure a useful tool for describing the level and development of socioeconomic differences in mortality and life expectancy over time.

2. **Result Section**

We also conducted detailed descriptive analyses to characterise mortality patterns across key demographic and socioeconomic groups. Using Stata’s survival‑time commands, we declared the data with stset and calculated crude mortality rates, person‑time at risk, and survival percentile. Descriptive estimates were obtained using the stsum, by(var) command, which tabulates the number of deaths, total person‑years, incidence rates, and Kaplan–Meier survival percentiles (25th, 50th, and 75th) within categories of each variable. Incidence rates were expressed as deaths per person‑year and, for interpretability, scaled to deaths per 1,000 person‑years. These descriptive statistics provided an unadjusted overview of mortality differences across SES, income, occupational class, migration background, health status, marital status, employment status, age groups, and overall life satisfaction. The result indicated that, despite comparable follow-up durations, men exhibited a higher crude mortality rate than women, with 8.4 deaths per 1,000 person-years compared with 6.2 per 1,000 person-years among women.

Marked social gradients in mortality were observed across indicators of SES position. Individuals in the low SES group had the highest crude mortality rate (7.1 per 1,000 person years), more than double that of the middle SES group (3.6 per 1,000) and over three times that of the high SES group (2.3 per 1,000). Similar patterns emerged across occupational class, income, and equivalised household income. Mortality rates were highest among those in low occupational classes (4.7 per 1,000 person years) and lowest in high occupational classes (2.6 per 1,000). Income differences were also pronounced: individuals in the low-income group had a mortality rate of 8.5 per 1,000, compared with 7.9 per 1,000 in the middle-income group and 4.0 per 1,000 in the high-income group. Using the Lambert equivalised income measure, mortality declined steadily from 8.4 per 1,000 in the lowest income category to 4.0 per 1,000 in the highest.

Migration background showed a distinct pattern: individuals without a migration background had a crude mortality rate of 8.4 per 1,000 person-years, whereas migrants exhibited a substantially lower rate of 3.8 per 1,000 person-years, consistent with a healthy migrant advantage.

Taken together, these descriptive findings demonstrate substantial and consistent social inequalities in mortality across SES, occupational class, income, and migration background. These unadjusted patterns closely parallel the gradients observed in the multivariable Cox models, underscoring the robustness of the socioeconomic disparities identified in this study. (See ***Supplementary Table S1)***.

***Supplementary Table S2.*** *Crude mortality rates, person‑time at risk, and survival percentiles by demographic and socioeconomic characteristics, SOEP 1996–2023****.***

| Variable | Category | Time at risk | Incidence rate | CMR per 1,000 PY | Individual (N) | Survival time (25%, 50%, 75%) |
| --- | --- | --- | --- | --- | --- | --- |
| Sex | Male | 325,433 | 0.0084472 | 8.4 | 54,988 | 73 / 83 / 91 |
|  | Female | 358,802 | 0.0062012 | 6.2 | 57,018 | 78 / 87 / 92 |
| Migration background | Non‑migrant | 515,116 | 0.0084039 | 8.4 | 68,719 | 76 / 85 / 92 |
|  | Migrant | 169,179 | 0.0038125 | 3.8 | 43,318 | 75 / 83 / 90 |
| Marital status | Married | 405,179 | 0.0067501 | 6.8 | 65,814 | 76 / 86 / 92 |
|  | Separated | 71,863 | 0.0073751 | 7.4 | 13,098 | 74 / 84 / 91 |
|  | Widowed | 37,071 | 0.0369831 | 37.0 | 5,819 | 74 / 83 / 91 |
|  | Single | 168,820 | 0.0019962 | 2.0 | 38,469 | 72 / 82 / 91 |
| Age group | 16–29 | 123,560 | 0.0004208 | 0.4 | 34,567 | — / — / — |
|  | 30–44 | 201,404 | 0.0007497 | 0.7 | 46,151 | — / — / — |
|  | 45–64 | 235,143 | 0.0046908 | 4.7 | 41,430 | — / — / — |
|  | 65+ | 124,188 | 0.0295359 | 29.5 | 18,761 | 79 / 86 / 92 |
| Education (CASMIN) | Low | 248,691 | 0.0128955 | 12.9 | 44,335 | 72 / 82 / 90 |
|  | Middle | 257,922 | 0.0038965 | 3.9 | 41,781 | 78 / 86 / 93 |
|  | High | 153,954 | 0.0043 | 4.3 | 25,745 | 81 / 89 / 96 |
| Occupational class | Low | 227,733 | 0.0046721 | 4.7 | 40,145 | 74 / 84 / 92 |
|  | Middle | 167,847 | 0.0031219 | 3.1 | 30,268 | 79 / 87 / 92 |
|  | High | 93,142 | 0.002566 | 2.6 | 17,577 | 18 / 81 / 91 |
| Income group (3 quantiles) | Low | 138,143 | 0.0085129 | 8.5 | 44,024 | 69 / 80 / 88 |
|  | Middle | 409,562 | 0.0079426 | 7.9 | 79,931 | 75 / 84 / 92 |
|  | High | 136,357 | 0.0039895 | 4.0 | 34,812 | 82 / 90 / 96 |
| SES-Index | Low | 84,084 | 0.0071 | 7.1 | 17,846 | 71 / 81 / 91 |
|  | Middle | 236,216 | 0.003645 | 3.6 | 38,512 | 76 / 86 / 93 |
|  | High | 71,461 | 0.002295 | 2.3 | 13,444 | 80 / 89 / 92 |
| Income group (5 quantiles) | <60% | 106,170 | 0.0083639 | 8.4 | 37,356 | 68 / 79 / 88 |
|  | 60–<80% | 118,155 | 0.0097499 | 9.7 | 41,850 | 71 / 81 / 89 |
|  | 80–<100% | 113,107 | 0.0086732 | 8.7 | 40,241 | 74 / 84 / 91 |
|  | 100–<150% | 189,977 | 0.0069482 | 6.9 | 50,717 | 77 / 86 / 93 |
|  | ≥150% | 156,886 | 0.0040348 | 4.0 | 38,911 | 82 / 90 / 96 |
| Study period | 1996–2005 | 193,662 | 0.0111173 | 11.1 | 34,645 | 70 / 79 / 86 |
|  | 2006–2014 | 228,636 | 0.0071336 | 7.1 | 52,380 | 77 / 86 / 93 |
|  | 2015–2023 | 261,997 | 0.004542 | 4.5 | 70,284 | 81 / 90 / 96 |
| **Birth cohort** | 1920–1939 | 75,254 | 0.0351078 | **35.1** | 8,691 | 73 / 83 / 93 |
|  | 1940–1954 | 129,402 | 0.0088329 | **8.8** | 13,952 | 78 / — / — |
|  | 1955–1969 | 198,750 | 0.0024453 | **2.4** | 26,421 | — / — / — |
|  | 1970–1984 | 176,175 | 0.0006755 | **0.7** | 30,699 | — / — / — |
| Employment status | Full‑time | 268,743 | 0.0015889 | 1.6 | 55,241 | 81 / — / — |
|  | Part‑time | 86,174 | 0.0011024 | 1.1 | 23,839 | —* / — / — |
|  | Vocational | 17,998 | 0.0005001 | 0.5 | 9,344 | — / — / — |
|  | Irregular | 33,275 | 0.0017731 | 1.8 | 14,147 | 86 / — / — |
|  | Not employed | 276,156 | 0.0158497 | 15.8 | 62,707 | 70 / 83 / 91 |
| SRH | Good | 570,303 | 0.003428 | 3.4 | 104,604 | 82 / 90 / 96 |
|  | Poor | 112,891 | 0.0265389 | 26.5 | 34,673 | 64 / 75 / 84 |
| Overall life satisfaction | Low | 27,596 | 0.0313451 | 31.3 | 13,589 | 60 / 69 / 76 |
|  | Medium | 155,990 | 0.011411 | 11.4 | 49,484 | 71 / 80 / 89 |
|  | High | 494,714 | 0.0045602 | 4.6 | 97,473 | 80 / 89 / 95 |

*Py= person-year, SRH = self-rated health, CMR= crude mortality rate per 1,000 persons‑years****, Incidence rate*** *= deaths / person‑time, *missing due to few events in cells,*

Kaplan–Meier and log-rank analyses examined unadjusted survival gradients by social group (SES), sex, and cohort. The overall survival curve followed the expected age-related pattern: survival probabilities remained near 1 in early adulthood, declined gradually through midlife, and fell sharply after age 70, approaching 0 at the maximum observed age of 104 years (Supplementary Figure S1).

***Supplementary Figure S1:*** *The Kaplan–Meier Survival Curve (overall)*

***Supplementary Figure S2:*** *The Kaplan–Meier Survival Curve SES-index with Gender (sex)*

**Supplementary Figure S3: The Kaplan–Meier Survival curve for Cohorts**

**Supplementary Table S3**. Cohort‑specific SES–mortality association (HR, 95% CI, p‑value)

| Cohort | Hazard Ratio (Low SES vs High SES) | 95% CI | p-value |
| --- | --- | --- | --- |
| 1920–1939 | 1.83 | 1.39–2.42 | 0.00 |
| 1940–1954 | 2.36 | 1.77–3.15 | 0.00 |
| 1955–1969 | 3.65 | 2.51–5.30 | 0.00 |
| 1970–1984 | 4.61 | 1.72–12.37 | 0.00 |

**Supplementary Figure S4**. Cohort‑specific estimates of the SES and mortality inequality

**Table S4**. Sensitivity Analysis: Cox Regression for SES and Period adjusted with Self‑Rated Health & overall Life Satisfaction

| Variable | Category | Hazard Ratio | 95% CI | p‑value |
| --- | --- | --- | --- | --- |
| **SES** | High (ref) | – | – | – |
|  | Low | **1.66** | 1.21–2.29 | 0.00 |
|  | Middle | **1.45** | 1.06–1.99 | 0.02 |
| **Period** | 1996–2005 (ref) | – | – | – |
|  | 2006–2014 | 0.69 | 0.47–1.01 | 0.05 |
|  | 2015–2023 | **0.53** | 0.36–0.78 | 0.00 |
| **SES × Period** | Low × 2006–2014 | 0.96 | 0.63–1.47 | 0.86 |
|  | Low × 2015–2023 | 1.28 | 0.82–2.01 | 0.28 |
|  | Middle × 2006–2014 | 0.97 | 0.64–1.47 | 0.89 |
|  | Middle × 2015–2023 | 0.94 | 0.61–1.44 | 0.76 |
| **Sex** | Female (ref) | – | – | – |
|  | Male | **1.84** | 1.66–2.05 | 0.00 |
| **Marital Status** | Married (ref) | – | – | – |
|  | Separated/Divorced | **1.23** | 1.06–1.42 | 0.01 |
|  | Widowed | 1.13 | 0.96–1.32 | 0.15 |
|  | Single | **1.64** | 1.35–2.00 | 0.00 |
| **Migration Background** | Non-migrant (ref) | – | – | – |
|  | Migrant | 0.94 | 0.82–1.08 | 0.36 |
| **Self‑Rated Health** | Good (ref) | – | – | – |
|  | Poor health | **2.93** | 2.62–3.27 | 0.00 |
| **Overall Life Satisfaction** | Low (ref) | – | – | – |
|  | Medium | **0.44** | 0.38–0.50 | 0.00 |
|  | High | **0.30** | 0.26–0.35 | 0.00 |

**Supplementary Table S5**. Sensitivity analysis: SES × cohort model adjusted for self‑rated health and overall life satisfaction

| Variable | Category | Hazard Ratio | 95% CI | p‑value |
| --- | --- | --- | --- | --- |
| **SES** | High (ref) | – | – | – |
|  | Low | 1.51 | 1.13–2.00 | 0.00 |
|  | Middle | 1.30 | 0.98–1.72 | 0.00 |
| **Cohort** | 1925–1939 (ref) | – | – | – |
|  | 1940–1954 | 0.64 | 0.44–0.92 | 0.02 |
|  | 1955–1969 | 0.36 | 0.24–0.56 | 0.00 |
|  | 1970–1984 | 0.15 | 0.06–0.38 | 0.00 |
| **SES × Cohort** | Low × 1940–1954 | 1.09 | 0.73–1.63 | 0.67 |
|  | **Low × 1955–1969** | **1.60** | **1.00–2.55** | **0.04** |
|  | Low × 1970–1984 | 2.29 | 0.82–6.36 | 0.11 |
|  | Middle × 1940–1954 | 1.04 | 0.71–1.54 | 0.83 |
|  | Middle × 1955–1969 | 1.13 | 0.73–1.77 | 0.58 |
|  | Middle × 1970–1984 | 2.46 | 0.94–6.39 | 0.06 |
| Sex | Female (ref) | – | – | – |
|  | Male | 1.85 | 1.66–2.06 | 0.00 |
| Marital Status | Married (ref) | – | – | – |
|  | Separated/Divorced | 1.20 | 1.03–1.39 | 0.01 |
|  | Widowed | 1.13 | 0.96–1.33 | 0.15 |
|  | Single | 1.64 | 1.35–1.99 | 0.00 |
| Migration Background | Non-migrant (ref) | – | – | – |
|  | Migrant | 0.92 | 0.80–1.06 | 0.27 |
| Self‑Rated Health | Good (ref) | – | – | – |
|  | Poor health | 2.93 | 2.62–3.28 | 0.00 |
| Overall Life Satisfaction | Low (ref) | – | – | – |
|  | Medium | 0.43 | 0.38–0.49 | 0.00 |
|  | High | 0.30 | 0.26–0.35 | 0.00 |

**Supplementary Table S6**. Sensitivity analysis: cumulative low SES adjusted for self-rated health and overall life satisfaction

| Variable | Category | HR | 95% CI | p‑value |
| --- | --- | --- | --- | --- |
| Cumulative low SES | Continuous | 1.10 | 1.05–1.16 | 0.00 |
| Sex | Female (ref) | – | – |  |
|  | Male | 1.73 | 1.63–1.84 | 0.00 |
| Marital status | Married (ref) |  |  |  |
|  | Separated/Divorced | 1.23 | 1.12–1.36 | 0.00 |
|  | Widowed | 1.34 | 1.24–1.45 | 0.00 |
|  | Single | 1.44 | 1.28–1.63 | 0.00 |
| Migration Background | Non-migrant (ref) | – | – |  |
|  | Migrant | 0.96 | 0.88–1.04 | 0.31 |
| Self‑Rated Health | Good (ref) | – | – | – |
|  | Poor health | 2.83 | 2.65–3.02 | 0.00 |
| Overall Life Satisfaction | Low (ref) | 2.95 | 2.70-3.22 | 0.00 |
|  | Medium | 1.54 | 1.44-1.65 | 0.00 |
|  | High (ref) | - | - |  |
| Time‑varying effect | cum_lowSES × age | 0.99 | 0.99–0.99 | 0.00 |

**Supplementary Figure S5: Cohort‑specific HR ratio low versus High SES**

**Detailed Sensitivity Analysis Report**

In the period and cohort models, socioeconomic inequalities in mortality persisted after adjustment, and the overall temporal and cohort patterns remained largely unchanged. This suggests that differences in subjective health and well-being do not fully explain the observed stability of SES-related mortality gradients. In the cumulative SES model, additional adjustment for health and well-being reduced the magnitude of the association between accumulated socioeconomic disadvantage and mortality, but did not eliminate it. A modest association persisted at age 50 and continued to decline with increasing age. For sensitivity models 1 and 2, the proportional hazards assumption was satisfied, and the overall pattern of results remained consistent with the main analyses, indicating that the findings are robust to alternative model specifications (see supplementary Tables S3, S4, and S5). To assess the robustness of the SES × period associations, we estimated an extended model additionally adjusted for self‑rated health and overall life satisfaction. These subjective health and well-being indicators were strong independent predictors of mortality, and their inclusion attenuated the SES coefficients, consistent with partial mediation. Nevertheless, both low and middle SES remained significantly associated with elevated mortality (Low SES: HR = 1.66, 95% CI 1.21–2.29; Middle SES: HR = 1.45, 95% CI 1.06–1.99). Mortality continued to decline across periods, with the strongest reduction observed in 2015–2023 (HR = 0.53, 95% CI 0.36–0.78).

None of the SES × period interaction terms reached statistical significance (all p > 0.28). This suggests that differences in subjective health or life satisfaction do not explain the stability of SES inequalities across historical periods. The proportional hazards assumption remained satisfied (global test p=0.14). The sensitivity analysis confirms that the main findings are robust: socioeconomic inequalities in mortality are substantial and persistent. Their relative magnitude does not change across study periods, even after accounting for subjective health and well-being. We also adjusted the SES × cohort model for self-rated health and life satisfaction to test the robustness of cohort-specific SES inequalities. As shown in Table 8, these indicators attenuated the magnitude of SES effects as expected. However, the overall pattern of SES–cohort differences remained similar. Notably, the interaction between low SES and the 1955–1969 cohort stayed statistically significant (HR=1.60, p=0.048). The interaction for middle SES in the youngest cohort showed a comparable effect direction. The proportional hazards assumption was satisfied for this fully adjusted model (global p=0.92), supporting the robustness of our findings. After further adjusting for self-rated health and life satisfaction, the association between cumulative low SES and mortality was reduced but not eliminated. At age 50, cumulative low SES had a modest direct association with mortality (HR=1.10, 95% CI 1.05–1.16), much smaller than in the main model. The time-varying coefficient indicated that this effect weakened with age (tvc HR=0.9987, p<0.001), echoing the age-related attenuation seen in the main model.

This pattern suggests mortality risk from cumulative socioeconomic disadvantage largely works through its long-term impact on health and psychosocial well-being. These factors absorb much of the total effect. Still, the small, age-dependent direct effect in mid-adulthood indicates that cumulative disadvantage continues to affect mortality risk even after accounting for these pathways.
